# Multimodal Integration of Ambulatory ECG and Clinical Features for Sudden Cardiac Death and Pump Failure Death Prediction^★^

**DOI:** 10.64898/2026.04.21.26351421

**Published:** 2026-04-22

**Authors:** Steven Swee, Irsyad Adam, Erika Yilin Zheng, Ethan Ji, Ding Wang, William Speier, Jeffrey Hsu, Kai-Wei Chang, Kalyanam Shivkumar, Peipei Ping

**Affiliations:** a Medical Informatics HA, University of California Los Angeles, USA; b Department of Physiology, University of California Los Angeles, USA; c Department of Computer Science, University of California Los Angeles, USA; d Department of Radiological Sciences, University of California Los Angeles, USA; e Department of Medicine / Cardiology Division, University of California Los Angeles, USA

**Keywords:** Multiple Instance Learning, TCN, LLM, Feature Fusion

## Abstract

Ambulatory electrocardiograms (ECG) provides continuous monitoring of the heart’s electrical activity. However, many existing machine learning and artificial intelligence models for analyzing ambulatory ECG traces are often unimodal and do not incorporate patient clinical context. In this study, we propose a multimodal framework integrating ambulatory ECG-derived representations with clinical text embeddings to predict two cardiac outcomes: sudden cardiac death and pump failure death. Ambulatory ECG traces are preprocessed, segmented, and encoded via a multiple instance learning and temporal convolutional neural network framework. In parallel, patient clinical features are parsed into structured prompts, which are passed through a large language model to generate clinical reasoning; this reasoning passes through a biomedical language encoder to generate a text embedding. With the ECG and text embeddings, we systematically evaluate multiple fusion strategies, including concatenation- and gating-based approaches, to integrate these two data modalities. Our results demonstrate that multimodal models consistently outperform unimodal baselines, with adaptive fusion mechanisms providing the greatest improvements in predictive performance. Decision curve analysis highlights the potential clinical utility of the proposed framework for risk stratification. Finally, we visualize model attention across modalities, including ECG attention patterns, segment-level saliency, heart rate variability features, and clinical reasoning, to contextualize patient-specific predictions.

## 1. Introduction

Sudden cardiac death (SCD) and pump failure death (PFD) are two major causes of mortality in heart failure [1, 2, 3]. Despite advances in pharmacologic therapy and medical devices, accurately identifying individuals at elevated risk for these outcomes remains a persistent clinical challenge [2, 3, 4, 5].

Ambulatory electrocardiograms (ECG), including extended Holter monitoring, supports continuous assessment of cardiac rhythm, capturing transient arrhythmias, ectopic burden, and intermittent conduction abnormalities [6, 7, 8]. These transient abnormalities carry important prognostic information for assessing the risk of SCD and PFD [2, 6, 7, 8]. However, translating ambulatory ECG recordings into reliable and clinically actionable predictors of long-term cardiac mortality remains complex and resource-intensive in routine practice [4, 9].

Machine learning (ML) and artificial intelligence (AI) models offer early promise in addressing these challenges. AI/ML models have demonstrated strong capability in characterizing waveform morphology (e.g., P waves, QRS duration, etc.) and identify arrhythmic events (e.g., atrial fibrillation, atrial flutter, etc.) [10, 11]. In parallel, large language models (LLMs) have demonstrated capability of reasoning over both structured and unstructured datasets [12, 13]. Domain-adapted medical LLMs can synthesize clinical features (e.g., demographics, medical history, laboratory values) and generate biomedical reasoning [14].

Despite these advancements, critical gaps remain. Many AI/ML models for ECG analysis are inherently unimodal, focusing primarily on identifying patterns within ambulatory ECG recordings without incorporating broader patient context [15, 16]. There has been mounting evidence that incorporation of patient clinical context with ECG interpretations yields more accurate diagnoses, both for physicians and AI/ML models [17, 18, 19, 20, 21]. Multimodal integration of time-series (ECG) and text/tabular (patient clinical features) data modalities is necessary to achieve a more comprehensive understanding of a patient’s condition.

To address these challenges, we introduce a multimodal framework that integrates ambulatory ECG recordings with clinical features. We have selected prediction of SCD and PFD as our use case. By structuring ECG-derived features into a coherent longitudinal representation and integrating them patient clinical features, our multimodal AI model learns unified patient-level representations for cardiac outcome prediction.

The main contributions of this paper are summarized as follows:

1. A multimodal framework that integrates ambulatory ECG-derived representations with clinical text embeddings to predict sudden cardiac death (SCD) and pump failure death (PFD).
2. A systematic evaluation of multimodal fusion strategies, including concatenation- and gating-based approaches, demonstrating that adaptive modality weighting improves predictive performance over unimodal models.
3. Visualization of model attention across ECG segments and LLM-generated reasoning, highlighting ECG markers and clinical features most predictive for SCD and PFD. These highlighted features align with known risk factors for SCD and PFD.

## 2. Related Works

### 2.1. Ambulatory ECG Analysis

Early computational studies subsequently emphasized time- and frequency-domain feature extraction to associate ambulatory ECG patterns with cardiac phenotypes. Badilini et al. [22] applied spectral analysis to evaluate circadian variation and beta-blockade effects, while Bakhshi et al. [23] used second-order autoregressive modeling and linear classification to distinguish arrhythmias from sinus rhythm.

More recent work has adopted deep learning to automate representation learning and rhythm classification. Huang et al. [24] transformed ECG segments into spectrograms and applied two-dimensional convolutional neural networks for rhythm classification, whereas Biton et al. [25] proposed Ar-Net2 to model temporal dependencies across RR intervals. Ben-Moshe et al. [26] introduced RawECGNet to address distributional shifts and detect atrial fibrillation directly from 30-second recordings. Han et al. [27] combined Gramian Angular Field representations with multiple instance learning for arrhythmia detection.

Across these approaches, ambulatory ECG recordings are typically analyzed as collections of shorter segments, with predictions generated at the segment level and, in some cases, aggregated through mechanisms such as multiple instance learning to obtain recording-level outputs. However, these strategies are limited to localized rhythm detection.

### 2.2. LLMs and Multimodal Integration

LLMs and domain-adapted language models have been studied for their utility in understanding clinical text and decision-support applications. Lee et al. introduced BioBERT [28], and Huang et al. developed ClinicalBERT [29], both leveraging domain-specific pretraining to adapt transformer architectures to biomedical and clinical corpora for improved encoding of clinical narratives and structured electronic health record data. More recently, Xie et al. proposed Me-LLaMA [14], and Li et al. introduced LLaVA-Med [30], exploring instruction tuning and multimodal alignment strategies to enable higher-level clinical reasoning, including summarization, question answering, and outcome prediction from heterogeneous patient information. Yang et al. [31] constructed ECG-LM, which processes both short-duration ECGs and clinical notes for classifying arrhythmic events and propose care strategies.

These approaches apply language models to textual data and, in some cases, short-duration ECG signals, primarily focusing on representation learning and arrhythmia classification tasks. To date, there has been limited work in integrating patient clinical context with 24-hour ambulatory ECG signals.

## 3. Methods

**Figure 1:**
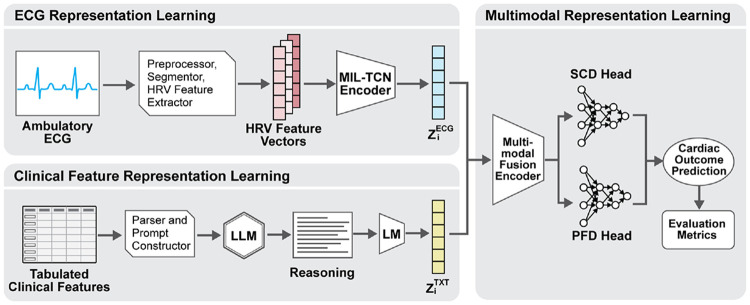
The Multimodal Workflow for Ambulatory ECG and Clinical Feature Analysis.

Fig. 1 illustrates our multimodal workflow for analyzing ambulatory ECGs together with clinical features. ECG recordings are preprocessed and segmented into short windows. For each window, heart rate variability (HRV) features are extracted and encoded via a Multiple Instance Learning–Temporal Convolutional Network (MIL–TCN), generating an ECG representation, $z_{i}^{\text{ECG}}$. In parallel, clinical variables are parsed into prompts and passed into an LLM the generated reasoning is encoded by a domain-adapted biomedical language model to generate a text representation, $z_{i}^{\text{TXT}}$. The ECG and text representations are fused within a multimodal fusion encoder to generate a unified patient embedding, which is used to predict SCD and PFD.

### 3.1. ECG Representation Learning

#### 3.1.1. ECG Preprocessing

All ambulatory ECG recordings undergo a standardized preprocessing pipeline for denoising. Signals were bandpass filtered between 0.5–40 Hz using a fourth-order Butterworth filter to attenuate baseline wander and high-frequency noise [32]. Baseline drift was further corrected via median filtering over a 0.8-second window, and the estimated baseline component was subtracted from the filtered signal [33]. R-peaks were then detected once per full-length recording using NeuroKit2 to ensure consistent beat localization across the entire ambulatory trace [34]. Full-record NeuroKit2 processing was subsequently performed to derive physiologic descriptors, including instantaneous heart rate, signal quality indices, and cardiac phases.

Following preprocessing, each recording was partitioned into non-overlapping 30-second segments. Segment start indices were precomputed to support efficient segmentation. Only complete windows were retained.

Within each segment, time-domain HRV metrics (e.g., SDNN, RMSSD, and pNN50) were computed. Rhythm descriptors such as premature ventricular contraction (PVC) burden, PVC couplets, and longest R–R pause were also computed. Additional NeuroKit2-based analysis was applied at the segment level to estimate interval measurements, including QRS duration and QT interval, as well as P- and T-wave peak counts. All segment-level features were stored as structured tabular representations for downstream modeling.

#### 3.1.2. Multiple Instance Learning with Temporal Convolutional Encoding

Under the multiple instance learning (MIL) paradigm [35, 36], each patient recording is represented as a bag of segment-level instances. Each patient is assigned binary outcome labels for each sudden cardiac death (SCD) and pump failure death (PFD), denoted by $y^{\text{SCD}}$, $y^{\text{PFD}}\in \{0,1\}$. The objective is to learn a mapping from segment-level representations to patient-level outcome probabilities.

For a given patient, the ambulatory ECG is partitioned into N non-overlapping 30-second segments, yielding a bag $𝒳={\left\{x_{n}\right\}}_{n=1}^{N}$, where $x_{n}\in R^{F}$ denotes the feature vector extracted from segment n and F is the number of HRV features. The number of segments N varies across patients depending on recording duration and signal quality.

Each segment $x_{n}$ is first projected into a learned embedding space using a shared feature encoder $f_{\theta }\;:R^{F}\to R^{d}$, producing segment embeddings $h_{n}=f_{\theta }(x_{n})$.

To model temporal dependencies across segments, the sequence of embeddings is processed by a dilated residual temporal convolutional network (TCN). Let ${\tilde{h}}_{n}^{\left(0\right)}=h_{n}$. Each residual block applies a dilated convolutional transformation followed by a residual connection:

(1) $${\tilde{h}}_{n}^{\left(l\right)}={\tilde{h}}_{n}^{\left(l-1\right)}+{𝒯}^{\left(l\right)}\left({\tilde{h}}_{n}^{\left(l-1\right)}\right),$$

where ${𝒯}^{\left(l\right)}$ denotes a dilated convolutional block with dilation factor $2^{l}$. The final output is denoted by ${\tilde{h}}_{n}={\tilde{h}}_{n}^{\left(L\right)}$.

To aggregate the variable-length sequence into a patient-level representation, we apply attention-based MIL pooling:

(2a) $$\alpha_{n}=\frac{\text{exp}(a({\tilde{h}}_{n}))}{\sum_{k=1}^{N}\text{exp}(a({\tilde{h}}_{k}))}$$

(2b) $$z_{i}^{\text{ECG}}=\sum_{n=1}^{N}\alpha_{n}{\tilde{h}}_{n}$$

where the attention score is computed as

$$a(h)=w_{2}^{T}\;\tanh(W_{1}h+b_{1})+b_{2},$$

For patient i, the ECG embedding $z_{i}^{\text{ECG}}\in R^{H}$ is passed to two task-specific branches for SCD and PFD prediction.

### 3.2. Clinical Feature Representation via Language Models

#### 3.2.1. Prompt Construction

We utilized two instruction-tuned large language models, LLaMA-3.1-8B-Instruct (LLaMA8B) and LLaMA-3.2-3B-Instruct (LLaMA3B) [37, 38], to generate clinical reasoning using patient clinical features. To construct the prompts, clinical features were parsed and converted into structured patient summaries describing demographics, comorbidities, medications, laboratory measurements, and summary ambulatory ECG impressions. Each prompt consisted of a system message defining the clinical persona and task constraints as well as a user message containing the patient-specific clinical features. The system message instructed the model to act as a cardiologist evaluating heart failure patients and to independently assess the risk of SCD and PFD. The model was required to produce categorical risk estimates (low, moderate, high) along with explanatory reasoning for each outcome. Example prompt templates are provided in Appendix A.

#### 3.2.2. Text Embedding Generation

Let the generated explanation for patient i be denoted as $t_{i}$. Each explanation is encoded using domain-adapted biomedical language models (i.e., BioBERT [28] and ClinicalBERT [29]) to produce a fixed-length embedding $e_{i}\in R^{p}$, obtained from the final hidden representation of the [CLS] token. These embeddings serve as patient-level text representations summarizing the clinical reasoning generated by the upstream LLM.

The embedding $e_{i}$ is then passed through a shared feature transformation $h_{\phi }\;:\;R^{p}\to R^{d_{t}}$ implemented as either a linear layer or a multi-layer perceptron with shared hidden layers, producing the patient-level representation $z_{i}^{TXT}=h_{\phi }(e_{i})$. The embedding $z_{i}^{TXT}$ is then passed to two task-specific branches corresponding to SCD and PFD prediction.

### 3.3. Multimodal Fusion Architecture

#### 3.3.1. Extraction of ECG and Text Embeddings

After training the unimodal models, we extract their respective patient representations. For the ECG modality, the trained MIL–TCN encoder is frozen and the patient-level embedding $z_{i}^{ECG}\in R^{d_{e}}$ obtained after attention-based pooling is extracted for each patient. Similarly, for the text modality, the trained text classifier is frozen and the shared representation $z_{i}^{TXT}\in R^{d_{t}}$ is extracted from the hidden layer preceding the task-specific prediction heads. Based on highest predictive performance, we selected two language–embedding configurations for the text modality: LLaMA8B–BioBERT and LLaMA3B–ClinicalBERT. Following the model selection criterion described in Section 3.4.2, the multimodal results presented in this work use the LLaMA8B–BioBERT configuration.

These modality-specific embeddings ($z_{i}^{ECG}$, $z_{i}^{TXT}$) serve as inputs to the multimodal fusion models described below.

#### 3.3.2. Multimodal Fusion Strategies

Given the ECG representation $z_{i}^{\text{ECG}}$ and text representation $z_{i}^{\text{TXT}}$ for patient i, we evaluate five multimodal fusion strategies to produce a fused representation $z_{i}^{F}$.

**Direct concatenation (DC)** concatenates raw embeddings without projection:

(3) $$z_{i}^{F}=[z_{i}^{\text{ECG}}\;∥\;z_{i}^{\text{TXT}}]$$

**Projected concatenation (PC)** first maps each modality into a shared latent space:

(4a) $$h_{i}^{\text{ECG}}=W_{e}z_{i}^{\text{ECG}}+b_{e},\;\;h_{i}^{\text{TXT}}=W_{t}z_{i}^{\text{TXT}}+b_{t}$$

(4b) $$z_{i}^{F}=[h_{i}^{\text{ECG}}\;∥\;h_{i}^{\text{TXT}}]$$

where $W_{e}$, $W_{t}$ are learned projection matrices.

**Scalar gating (SG)** combines projected representations via a single learnable gate:

(5) $$z_{i}^{F}=g\;h_{i}^{\text{ECG}}+(1-g)h_{i}^{\text{TXT}},\;\;g=\sigma (\alpha )$$

where α∈R is a global scalar parameter shared across all patients.

**Vector gating (VG)** computes a patient-specific gating vector conditioned on both modalities:

(6a) $$g_{i}=\sigma (W_{g}[h_{i}^{\text{ECG}}\;∥\;h_{i}^{\text{TXT}}]+b_{g})$$

(6b) $$z_{i}^{F}=g_{i}⊙h_{i}^{\text{ECG}}+(1-g_{i})⊙h_{i}^{\text{TXT}}$$

In all cases, $z_{i}^{F}$ is passed to two task-specific prediction branches for SCD and PFD.

### 3.4. Model Training and Optimization

#### 3.4.1. Loss Functions

All models are trained using a multi-task objective to jointly predict SCD and PFD. For each task, we use weighted binary cross-entropy loss, with the positive-class weight set to the negative-to-positive ratio in the training fold, $w_{t}=N_{\text{neg}}∕N_{\text{pos}}^{t}$, to account for class imbalance. The overall training objective sums the two task-specific losses:

(7) $$ℒ={ℒ}_{\text{SCD}}+{ℒ}_{\text{PFD}}$$

Models are trained patient-by-patient (batch size of one), with losses computed on a single patient’s fused representation per update step.

#### 3.4.2. Training Protocol and Cross-Validation

All experiments were conducted on a Linux server running Ubuntu and equipped with NVIDIA GeForce RTX 2080 Ti GPUs. Models were implemented in PyTorch (v2.5.1) with CUDA support (CUDA 12.4) for GPU acceleration. Model training was performed using stratified 5-fold cross-validation and hyperparameter tuning was conducted within this framework. Details of the grid search parameter space are shown in Appendix B. The ECG encoder was trained for 30 epochs, while the text classifiers and multimodal fusion models were trained for 100 epochs.

Model selection was based on the highest mean area under the receiver operating characteristic curve (AUC). The mean AUC was computed as the average of the AUC for SCD and PFD, which were weighted equally due to their near-identical prevalence in the dataset (9.92% and 11.66% respectively). Final performance metrics were reported across the five cross-validation folds.

## 4. Experiments

### 4.1. Dataset

Experiments were conducted with the publicly available dataset from MUSIC (MUerte Subita en Insuficiencia Cardiaca) study, a prospective multicenter cohort of patients with chronic heart failure [39, 40, 41]. The study enrolled 992 patients from eight Spanish university hospitals between April 2003 and December 2004, with a median follow-up of 44 months. For each subject, a 24-hour Holter electrocardiogram (ECG) was acquired using SpiderView recorders (ELA Medical, Sorin Group, Paris, France) with two or three orthogonal leads (X, Y, Z) sampled at 200 Hz with 10 μV amplitude resolution. These Holter recordings constitute the ambulatory ECG signals analyzed in this work.

Following the initial cohort definition, cohort selection criteria were applied. Individuals with prior implantable cardiac devices (e.g., pacemakers), patients who exited the study due to cardiac transplantation, or patients with unknown outcomes were excluded. The final cohort consisted of 746 patients.

For signal analysis, only Lead Y was used in this study, which corresponds to the superior–inferior electrical axis and has similar orientation to Lead II in the standard 12-lead ECG. This alignment makes it suitable for beat detection and heart rate variability (HRV) analysis using the NeuroKit2 toolbox [34, 42].

### 4.2. Evaluation Metrics

Model performance was evaluated using five-fold cross-validation. Patients were partitioned into five mutually exclusive folds, ensuring that all segments from a given patient remained within the same fold. For each iteration, four folds were used for training and one fold for validation. Performance metrics were computed on the held-out fold and averaged across all folds [43]. Evaluation was performed separately for two prediction tasks: survivor versus SCD and survivor versus PFD.

Let TP, TN, FP, and FN denote the number of true positives, true negatives, false positives, and false negatives, respectively. The following metrics were used to evaluate model performance [44]:

(8) $$\text{Accuracy}=\frac{TP+TN}{TP+TN+FP+FN}$$

(9) $$\text{Precision}=\frac{TP}{TP+FP}$$

(10) $$\text{Recall}=\frac{TP}{TP+FN}$$

(11) $$F1=\frac{2\cdot \text{Precision}\cdot \text{Recall}}{\text{Precision}+\text{Recall}}$$

In addition to threshold-based metrics, the area under the receiver operating characteristic curve (AUC) was computed to evaluate discrimination performance across all classification thresholds. To assess potential clinical utility, decision curve analysis (DCA) was also performed. DCA evaluates the net benefit of a predictive model across a range of decision thresholds, allowing comparison between the proposed model and alternative strategies such as treating all patients or treating none [45].

### 4.3. Experimental Results

**Figure 2:**
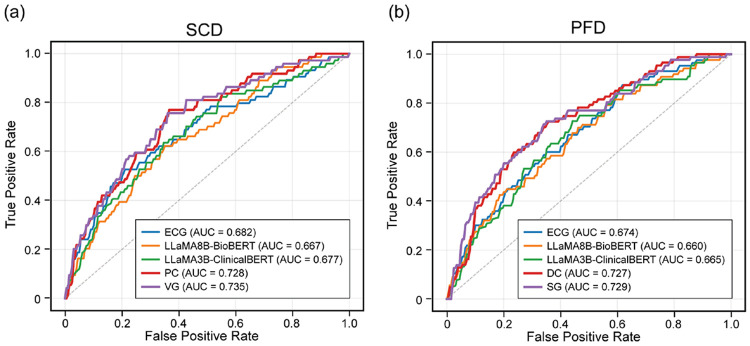
Comparison of AUC curves for unimodal (ECG, LLaMA8B-BioBERT, LLaMA3B-ClinicalBERT) and multimodal (PC, VG, DC, and SG) models for sudden cardiac death (a) and pump failure death (b).

**Figure 3:**
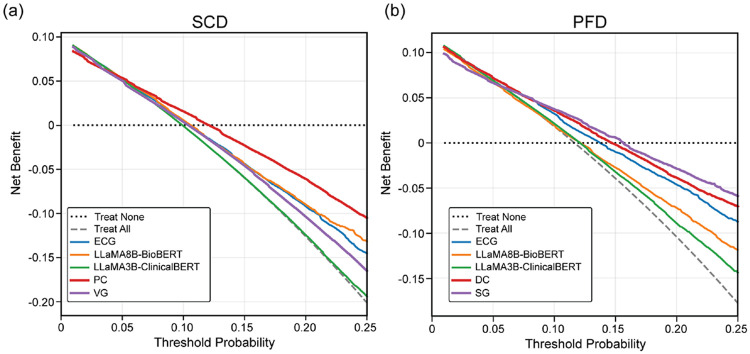
Comparison of DCA curves for unimodal (ECG, LLaMA8B-BioBERT, LLaMA3B-ClinicalBERT) and multimodal (PC, VG, DC, and SG) models for sudden cardiac death (a) and pump failure death (b).

**Figure 4:**
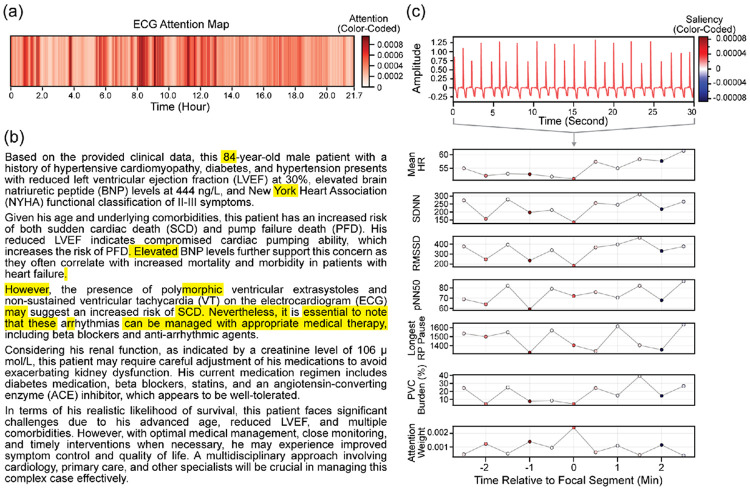
Attention map for the ambulatory ECG (a), attention map for the LLM response (b), as well as saliency maps for a select ECG segment and HRV features (c). These maps are for the vector gating multimodal model in which the patient predicted to have high risk of sudden cardiac death.

**Figure 5:**
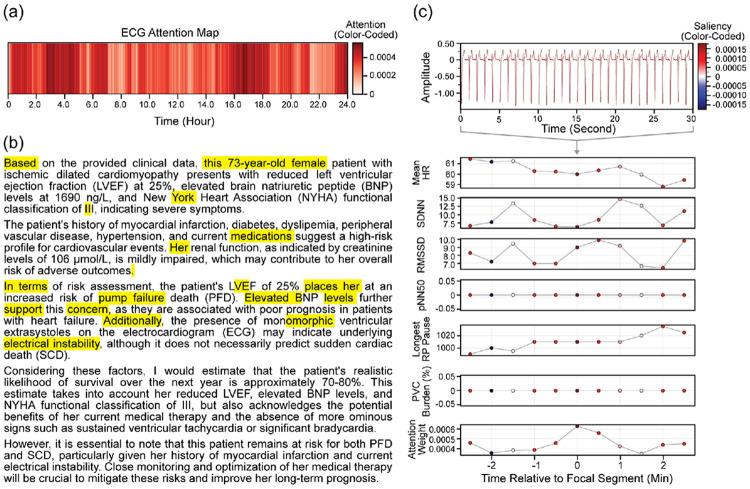
Attention map for the ambulatory ECG (a), attention map for the LLM response (b), as well as saliency maps for a select ECG segment and HRV features (c). These maps are for the direct concatenation multimodal model in which the patient predicted to have high risk of pump failure death.

The performance metrics for SCD and PFD prediction for both unimodal and multimodal models are summarized in Table 1 and Table 2, respectively. Corresponding AUC curves and DCA plots are shown in Fig. 2 and Fig. 3. In addition to quantitative evaluation, Fig. 4 and Fig. 5 visualize multimodal model attention across ECG segments and clinical reasoning for representative patient cases.

#### 4.3.1. Unimodal Model Performance

First, we evaluated models trained independently on ambulatory ECG features and text-derived clinical representations. Across both prediction tasks, the ECG-only model demonstrated the strongest discrimination performance. For SCD prediction, the ECG-only model achieved an AUC of 0.682 ± 0.030, outperforming text-only configurations. Text-based models achieved slightly lower discrimination performance overall, with AUC values ranging from 0.627 to 0.677. A similar pattern was observed for PFD prediction. The ECG-only model achieved an AUC of 0.674 ± 0.100, while text-based models achieved AUC values between 0.616 and 0.665.

#### 4.3.2. Multimodal Fusion Performance

Next, we evaluated several multimodal fusion strategies integrating ECG and text representations, including direct concatenation (DC), projected concatenation (PC), scalar gating (SG), and vector gating (VG).

For SCD prediction, multimodal fusion consistently improved discrimination performance compared with unimodal models. Multimodal models achieved AUC values ranging from 0.702 to 0.735, with the vector gating model achieving the highest AUC of 0.735 ± 0.055, followed closely by the projected concatenation model with an AUC of 0.728 ± 0.041. These results represent an improvement of approximately 0.05 AUC compared with the ECG-only baseline. F1 scores were comparable between ECG-only and multimodal models. These differences suggest that the ECG-only model exhibits a more liberal classification pattern, achieving higher recall, whereas multimodal models are more conservative, achieving higher precision.

A similar trend was observed for PFD prediction. Multimodal models achieved AUC values ranging from 0.704 to 0.729, with the scalar gating model achieving the highest AUC of 0.729 ± 0.078, followed closely by the direct concatenation model with an AUC of 0.727 ± 0.074. As with SCD, the ECG-only model demonstrated relatively high recall, whereas multimodal models achieved higher precision. F1 scores for multimodal models meet or exceed the F1 score for the ECG-only model by a standard deviation.

Collectively, these findings demonstrate that integrating ambulatory ECG-derived physiological features with language-derived clinical representations improves discrimination while shifting model behavior toward higher precision and more balanced classification performance across both cardiac outcomes.

#### 4.3.3. Decision Curve Analysis

Decision curve analysis was performed to evaluate the potential clinical utility of the proposed models. As shown in Fig. 3, the multimodal models generally demonstrate higher net benefit compared with unimodal approaches across a range of clinically relevant decision thresholds.

For SCD prediction, projected concatenation exhibits improved net benefit relative to unimodal baselines, although the vector gating model shows comparable benefit with the unimodal models. For PFD prediction, both direct concatenation and scalar gating models consistently demonstrate greater net benefit compared with unimodal approaches. These findings suggest that integrating ambulatory ECG-derived physiological features with language-derived clinical representations may provide improved clinical decision support across a range of risk thresholds.

#### 4.3.4. Multimodal Model Interpretability

To illustrate model behavior, we present visualizations of multimodal attention for representative patients predicted to experience SCD and PFD in Fig. 4 and Fig. 5, respectively. Computed attention weights over the full ambulatory ECG (Fig. 4a and Fig. 5a) show intermittent, higher-weight regions, rather than a single isolated event. These regions suggest that the model relies on recurring or transient events distributed throughout the recording, indicating that risk for SCD and PFD is associated with repeated temporal patterns rather than a single dominant episode. For the attention patterns in the LLM-generated clinical reasoning (Fig. 4b and Fig. 5b), higher attention is assigned to specific clinical descriptors. In both cases, demographic (e.g., age and gender), references to medications and clinical management, and transitional phrases such as “additionally,” “however,” and “in terms of” are emphasized, indicating that the model attends to multiple elements within the structured clinical reasoning.

To examine local model behavior, representative 30-second ECG segments are shown alongside segment-level HRV features, colored by saliency weights (Fig. 4c and Fig. 5c). In the SCD example, segments with higher attention weights are associated with modest increases in mean heart rate and relatively lower short-term variability (e.g., lower RMSSD), with low pNN50 and minimal PVC burden. These patterns reflect subtle variability changes rather than overt arrhythmic events. In contrast, the PFD example shows elevated attention in segments characterized by relatively higher heart rate (approximately 60 bpm) and reduced variability (e.g., lower SDNN and RMSSD). Additionally, the longest RR pause increases toward later segments in the PFD case, aligning with regions of higher attention. Across both cases, attention highlights segments prioritized by the model during temporal aggregation, linking ECG-derived features with clinical reasoning at the patient level.

## 5. Discussion

We systematically evaluated multimodal fusion strategies that integrate ambulatory ECG-derived representations with clinical text embeddings. Integrating these data modalities led to improved prediction for both SCD and PFD compared to unimodal approaches. While ECG-derived representations provided the strongest standalone predictive signal, the addition of text embeddings yielded measurable gains in performance by incorporating patient-specific clinical context that helps distinguish similar ECG patterns with different underlying risk profiles. Ambulatory ECG recordings capture continuous rhythm patterns and transient events, whereas clinical text encodes complementary information such as demographics and medical history. Fusion mechanisms, particularly gating-based approaches, enable adaptive weighting of these modalities at the patient level, allowing the model to prioritize the most informative source of information for each case.

Beyond predictive performance, we present patient-level visualization of model attention across modalities. By linking attended ECG segments to corresponding heart rate variability features and clinical reasoning, the model highlights the features contributing to its predictions. These visualizations offer insight into how information is integrated across time-series and clinical context, supporting more transparent interpretation of model behavior in clinical settings.

Several limitations should be considered. First, the study is conducted on a single dataset; external validation is necessary to assess generalizability. Second, the relatively limited number of SCD and PFD events may constrain model robustness, particularly for rare outcome prediction. Third, the current framework relies on engineered HRV features rather than fully end-to-end raw signal modeling, which may limit the capture of more complex waveform-level information. Additionally, the quality and consistency of language model–generated reasoning may introduce variability in text representations. Future work will address these limitations by expanding validation to independent cohorts, integrating raw ECG signal representations through end-to-end deep learning architectures, and improving alignment between time-series and language-based embeddings via joint training.

## 6. Conclusion

We present a multimodal AI framework that leverages ambulatory ECG traces with clinical features to predict sudden cardiac death and pump failure death. Combining both physiological signals with clinical context led to improved predictions compared to unimodal approaches. Further, attention maps offer explainability by highlighting features that most contribute to cardiac outcome predictions. Our findings highlight the synergistic roles of signal-level electrophysiology and clinical reasoning in cardiac outcome prediction. This framework can be extended to additional cardiovascular outcomes and supports the development of interpretable, multimodal AI approaches for patient-specific risk assessment.

## Figures and Tables

**Table 1:** Comparison of unimodal and multimodal models for sudden cardiac death prediction. Results are reported as mean ± standard deviation across five-fold cross-validation.

Model	AUC	Accuracy	Precision	Recall	F1
Unimodal					
ECG Only	0.682±0.030	0.718±0.080	0.196±0.061	**0.540±0.060**	0.284±0.067
LLaMA8B-BioBERT	0.667±0.074	0.749±0.097	0.141±0.107	0.409±0.295	0.208±0.154
LLaMA3B-BioBERT	0.627±0.064	0.764±0.095	0.199±0.074	0.390±0.140	0.253±0.083
LLaMA8B-ClinicalBERT	0.640±0.062	0.764±0.131	0.132±0.086	0.289±0.243	0.164±0.096
LLaMA3B-ClinicalBERT	0.677±0.035	0.673±0.137	0.119±0.070	0.490±0.300	0.191±0.113
Multimodal					
DC	0.701±0.060	0.732±0.034	0.179±0.024	0.475±0.110	0.258±0.033
PC	0.728±0.041	0.740±0.029	0.193±0.029	0.514±0.156	0.277±0.053
SG	0.712±0.054	**0.767±0.056**	**0.212±0.032**	0.475±0.152	**0.285±0.041**
VG	**0.735±0.055**	0.763±0.065	0.207±0.036	0.473±0.142	0.282±0.053

**Table 2:** Comparison of unimodal and multimodal models for pump failure death prediction. Results are reported as mean ± standard deviation across five-fold cross-validation.

Model	AUC	Accuracy	Precision	Recall	F1
Unimodal					
ECG Only	0.674±0.100	0.622±0.063	0.169±0.035	0.589±0.231	0.260±0.063
LLaMA8B-BioBERT	0.660±0.075	0.737±0.120	0.157±0.095	0.368±0.249	0.210±0.125
LLaMA3B-BioBERT	0.660±0.087	0.753±0.046	0.190±0.037	0.383±0.287	0.242±0.088
LLaMA8B-ClinicalBERT	0.616±0.054	0.771±0.126	0.109±0.117	0.231±0.212	0.145±0.145
LLaMA3B-ClinicalBERT	0.665±0.104	0.761±0.115	0.121±0.115	0.359±0.356	0.180±0.173
Multimodal					
DC	0.727±0.074	0.725±0.055	0.231±0.042	0.586±0.214	**0.327±0.067**
PC	0.720±0.066	0.698±0.102	0.231±0.052	**0.621±0.220**	0.324±0.070
SG	**0.729±0.078**	**0.776±0.073**	**0.285±0.090**	0.495±0.265	0.325±0.064
VG	0.717±0.066	0.744±0.115	0.220±0.072	0.404±0.278	0.258±0.063

**Figure A.1:**
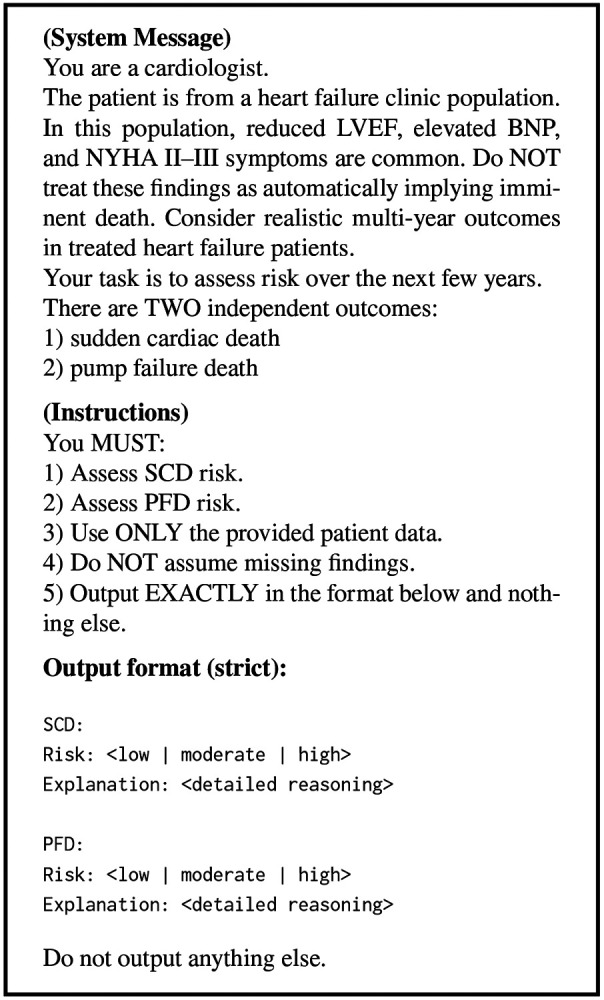
Prompt template for System Message and Instructions.

**Figure A.2:**
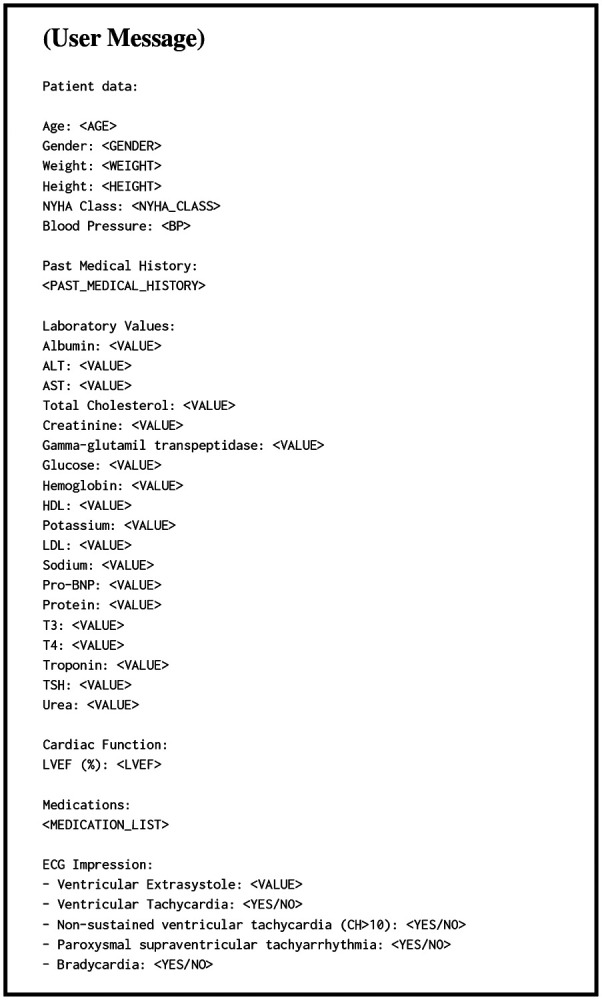
Prompt template for parsed patient clinical features.

## Data Availability

Data used in this paper are available on the MIT Physionet database at: https://physionet.org/content/music-sudden-cardiac-death/1.0.1/. Source code will be made publicly available upon publication.

## Appendix

### B. Hyperparameter Selection

We present the hyperparameters tested in Table B.1, Table B.2, and Table B.3.

**Table B.1:** Hyperparameters tested for ECG-only (MIL-TCN encoder) classifier.

Hyperparameters	Values Tested
Embedding Dimension	128, 256
TCN Hidden Dimension	128, 256
TCN Number of Hidden Layers	3, 4
Encoder Dropout	0.0, 0.2
TCN Dropout	0.1, 0.3
Branch Dropout	0.0, 0.2
Learning Rate	3e-5, 1e-4, 3e-4, 1e-3
Weight Decay	0.0, 1e-5

**Table B.2:** Hyperparameters tested for the text embedding classifier.

Hyperparameters	Values Tested
MLP Number of Layers	0, 1, 2, 3
MLP Hidden Layer Dimension	64, 128, 256, 512
Dropout	0.0, 0.1, 0.2, 0.3, 0.4, 0.5
Learning Rate	5e-3, 1e-3, 5e-4, 1e-4
Weight Decay	0, 1e-6, 1e-5, 1e-4, 1e-3

**Table B.3:** Hyperparameters tested for multimodal classifier.

Hyperparameters	Values Tested
Projection Dimension	128, 256
MLP Number of Hidden Layers	1, 2, 3
MLP Hidden Dimension	64, 128, 256
Dropout	0.0, 0.2, 0.4
Learning Rate	1e-3, 3e-4, 1e-4
Weight Decay	0, 1e-5
